# Genetic structure of *Mycoplasma ovipneumoniae* informs pathogen spillover dynamics between domestic and wild Caprinae in the western United States

**DOI:** 10.1038/s41598-019-51444-x

**Published:** 2019-10-25

**Authors:** Pauline L. Kamath, Kezia Manlove, E. Frances Cassirer, Paul C. Cross, Thomas E. Besser

**Affiliations:** 1 School of Food and Agriculture, University of Maine, Orono, ME 04469 USA; 2 U.S. Geological Survey, Northern Rocky Mountain Science Center, Bozeman, MT 59715 USA; 3 Department of Veterinary Microbiology and Pathology, Washington State University, Pullman, WA 99164 USA; 4 Department of Wildland Resources and Ecology Center, Utah State University, Logan, UT 84322 USA; 5 Idaho Department of Fish and Game, Lewiston, ID 83501 USA

**Keywords:** Ecological epidemiology, Ecological genetics, Phylogenetics

## Abstract

Spillover diseases have significant consequences for human and animal health, as well as wildlife conservation. We examined spillover and transmission of the pneumonia-associated bacterium *Mycoplasma ovipneumoniae* in domestic sheep, domestic goats, bighorn sheep, and mountain goats across the western United States using 594 isolates, collected from 1984 to 2017. Our results indicate high genetic diversity of *M. ovipneumoniae* strains within domestic sheep, whereas only one or a few strains tend to circulate in most populations of bighorn sheep or mountain goats. These data suggest domestic sheep are a reservoir, while the few spillovers to bighorn sheep and mountain goats can persist for extended periods. Domestic goat strains form a distinct clade from those in domestic sheep, and strains from both clades are found in bighorn sheep. The genetic structure of domestic sheep strains could not be explained by geography, whereas some strains are spatially clustered and shared among proximate bighorn sheep populations, supporting pathogen establishment and spread following spillover. These data suggest that the ability to predict *M. ovipneumoniae* spillover into wildlife populations may remain a challenge given the high strain diversity in domestic sheep and need for more comprehensive pathogen surveillance.

## Introduction

Disease management at the interface between wildlife and livestock is crucial for animal health and conservation, but remains a logistical and scientific challenge^1^. The role of a given species as a reservoir versus spillover host is particularly difficult to determine^2–4^ as long-term surveillance data are often lacking, which limits the inferences that can be made about the amount of disease transmission occurring within versus across species. However, genetic data from pathogens have recently proven valuable for gaining insights into pathogen spillover and transmission between livestock and wildlife^5–9^. In this study, we investigated the genetic relationships of the respiratory pathogen, *Mycoplasma ovipneumoniae*, among domestic and wild sheep and goats across the western United States to elucidate pathogen transmission dynamics.

Bronchopneumonia has been a key contributor to the historical declines and widespread extirpations of bighorn sheep (*Ovis canadensis*) across their range in western North America^10^. The disease is believed to have originated from pathogen transmission to bighorn sheep following exposure to domestic sheep (*Ovis aries*) and goats (*Capra hircus*) accompanying European settlers as they expanded westward^11^. As a result of disease-related die-offs, overharvesting, and habitat loss and fragmentation, the range-wide population dramatically decreased in size from a rough estimate of 1.5-2 million sheep in the early 1800s to under 40,000 sheep in the United States by the end of the 19^th^ century^12^. Today, the disease continues to severely limit recruitment, abundance, and distribution of the bighorn sheep^13–15^, impeding conservation efforts to reestablish the species across its range.

Bronchopneumonia of bighorn sheep is a complex polymicrobial disease with an etiology that has been extensively debated in the scientific literature. While multiple bacterial species, including *Mannheimia haemolytica*, *Pasteurella multocida*, and *Fusobacterium necrophorum*, have been detected in the lungs of affected individuals, amassed evidence points to *Mycoplasma ovipneumoniae* as the primary causative agent of pneumonia epizootics in bighorn sheep^10,16^. Furthermore, *M. ovipneumoniae* has been identified as cause of pneumonia outbreaks in other wild Caprinae species, including both free-ranging Norwegian muskox (*Ovibos moschatus*)^17^ and captive Dall’s sheep (*Ovis dalli dalli*)^18^.

Domestic Caprinae hosts, particularly domestic sheep, are thought to be a reservoir and source of pathogen infection to naïve bighorn sheep populations. The prevalence of *M. ovipneumoniae* was high (60%) in a sample of domestic sheep studied as part of the 2011 National Animal Health Monitoring Survey^19,20^. Domestic sheep as a reservoir of infection has also been supported by field observations of pneumonia-related bighorn mortalities following association with domestic sheep^21^. In addition, across 12 experimental commingling trials, ~99% of bighorn sheep died from pneumonia after contact with domestic sheep, together providing convincing evidence that contact with domestic sheep is a key risk factor for lethal pneumonia outbreaks in bighorn sheep^22^. A smaller set of experiments have also shown that domestic goats are capable of transmitting the pathogen to bighorn sheep; however the resulting respiratory disease symptoms were of reduced severity, with no fatalities observed^23^.

A variety of factors, including behavior^24–26^, herd density^27,28^, and social structure^29^ may influence the risk of pathogen exposure and transmission in wild sheep populations. In domestic sheep, operation size and management type were associated with the probability of *M. ovipneumoniae* infection, with larger and unfenced herded operations at higher risk^20^. The primary mechanism by which some of these factors likely influence pathogen spillover and transmission risk is through alterations in the spatial overlap, and thus contact rates, of wild and domestic hosts^28^ Therefore, to reduce this risk, federal and state natural resource agencies have implemented policies focused on the spatial separation of wild sheep and domestic Caprinae^30^.

Pathogen persistence and spread may also involve both natural and anthropogenic movement of wildlife. Translocation, in particular, has been extensively used as an approach for restoring bighorn sheep across their former range and, in some cases, has been successful in increasing population abundance and genetic diversity^31–34^. However, translocations may also introduce pathogens into naïve populations^35^. Bighorn sheep are a spatially-structured species, with loosely connected populations that reside in steep, rugged terrain. During an epizootic event, this structure may help to localize intraspecific pathogen transmission by limiting contact between neighboring populations or subpopulations^29^. However, rams have been shown to occasionally move more than 30 km beyond their core herd home range^25^, which may facilitate pathogen introductions into previously uninfected herds. Here, we assess what the genetics of *M. ovipneumoniae* can tell us about broad scale pathogen movement within and across host species.

**Figure 1:**
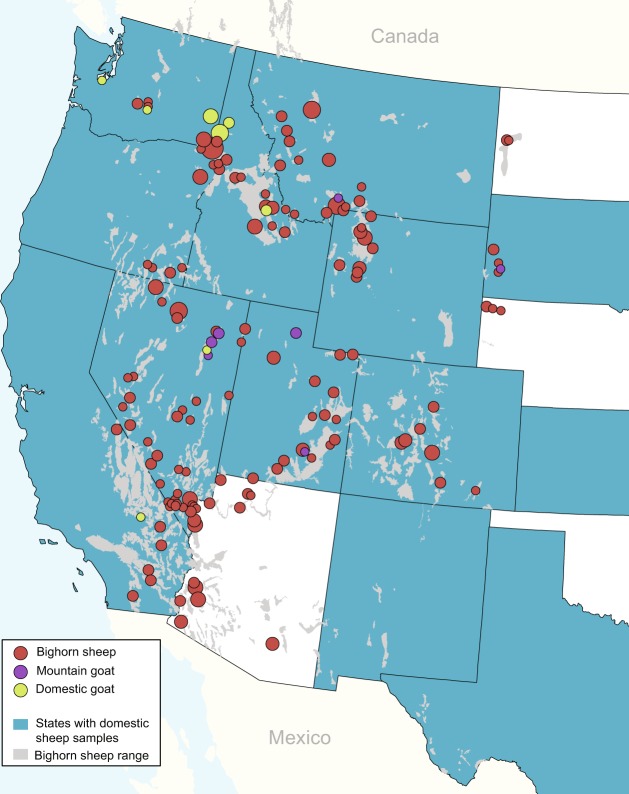
*Mycoplasma ovipneumoniae* isolate locations and host species. Samples were derived from bighorn sheep (red; *n* = 349), mountain goat (purple; *n* = 12), domestic goat (green; *n* = 34), and domestic sheep (blue; *n* = 208). States from which domestic sheep samples were obtained are represented by shading, rather than points, as state localizations are confidential. Isolates from Michigan, Minnesota, Wisconsin, China, and Australia are not shown. Circle size is relative to isolate sample size from a particular host and location. Current occupied bighorn sheep habitat distribution is shown in gray (Wild Sheep Working Group).

Knowledge on the pathways of *M. ovipneumoniae* transmission is lacking, particularly at landscape-level spatial scales. We examined the strain diversity and phylogeographic structure of *M. ovipneumoniae* in domestic and wild Caprinae hosts affected by bronchopneumonia across the western United States (Fig. 1). Our primary objectives were to (1) evaluate *M. ovipneumoniae* transmission within and among hosts and locations, and (2) evaluate patterns of pathogen spillover and persistence in bighorn sheep populations. These results elucidated broad-scale *M. ovipneumoniae* transmission dynamics, data that may inform disease control strategies to promote bighorn sheep conservation.

## Results

### Strain diversity in wild and domestic sheep and goats

We found a remarkably high number of *M. ovipneumoniae* strains (each defined as a group of sequence variants that differed by no more than 4 base pairs in pairwise comparisons) in domestic sheep flocks, with a total of 184 strains in 207 sheep sampled in the U.S (Dataset 1). The vast majority (159 out of 207, 77%) of individual domestic sheep possessed unique strains, and a single strain was never detected in more than three sheep. Only three strains (DS-7, DS-22, DS-23) were detected in more than a single operation, each in two domestic sheep operations located in different states. Otherwise herd strain composition was 100% divergent between any two operations. Although fewer samples were obtained from domestic goats in the U.S., a relatively high number of strains were observed, with a total of 16 strains in 26 individual goats. A moderate proportion of goats also had unique strains (9 out of 26, 35%); one strain (DG-3) was shared among three operations in WA, and another (DG-6) between three operations located in two different states (WA, NV).

In contrast, 88 strains were identified in 349 bighorn sheep (Dataset 1). Only 9% of individual bighorn sheep had unique strains, and 82% of bighorn sheep possessed a strain that was shared with at least two other bighorn sheep in our dataset. Of the approximately 134 bighorn sites sampled, we observed more than one strain at any given site only 35 times (26% of sites). Two strains detected in bighorn sheep were shared with domestic species: one strain from domestic sheep (BHS-55/DS-96) and one from domestic goats (BHS-50/DG-6). In the 12 mountain goats sampled, there were 5 strains, three of which were also found in bighorn sheep (MTG-1/BHS-48, MTG-4/BHS-32, MTG-5/BHS-37).

**Figure 2:**
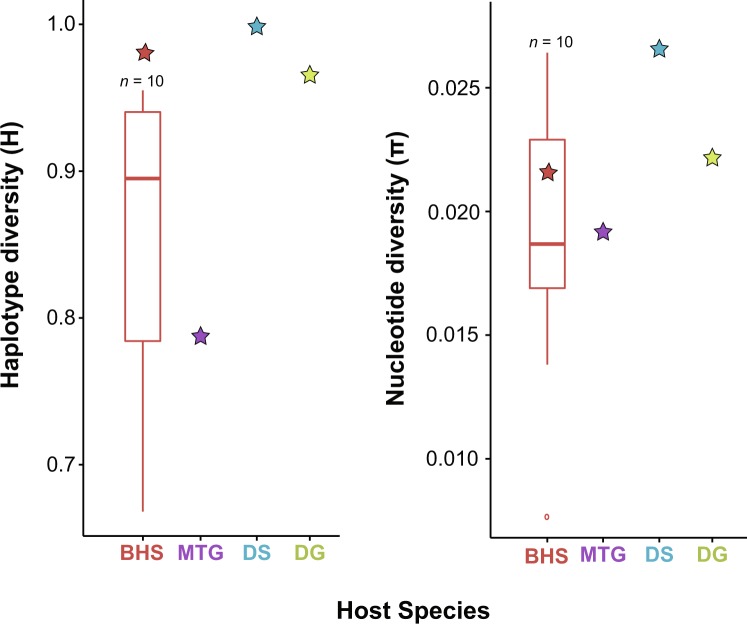
Haplotype and nucleotide diversity estimates in bighorn sheep (BHS; red), showing variation in estimates made at the state-level (boxplot; *n* = 10 states) and over all samples (star). Overall diversity estimates are shown for other Caprinae host species, including mountain goat (MTG; purple), domestic sheep (DS; blue), and domestic goat (DG; green), and are also reported in Table S2. Diversity was not estimated at the state-level for domestic sheep, domestic goats, and mountain goats due to lack of available state-level information or sufficient samples from more than one state. See Tables S3, S4 for the geographic distribution of the data by host and state.

Despite a smaller sample size, all estimated genetic diversity indices at the national level were higher in domestic sheep (*n* = 179, *A* = 162, *H*_d_ = 0.999, *π* = 0.027) than in bighorn sheep (*n* = 341, *A* = 118, *H*_d_ = 0.981, *π* = 0.022) (Fig. 2, Table S2). Diversity was similarly high across regional groupings of domestic sheep (Table S3). *M. ovipneumoniae* genetic diversity in domestic goats was lower than that observed in domestic sheep (*n* = 26, *A* = 17, *H*_d_ = 0.966, *π* = 0.022), and comparable to levels of diversity in bighorn sheep (Fig. 2, Table S2). In contrast, *M. ovipneumoniae* genetic diversity was low in mountain goats (*n* = 12, *A* = 5, *H*_d_ = 0.788, *π* = 0.019), falling within the range of state-level bighorn sheep diversity estimates (Fig. 2, Table S4).

### Rarefaction analyses

**Figure 3:**
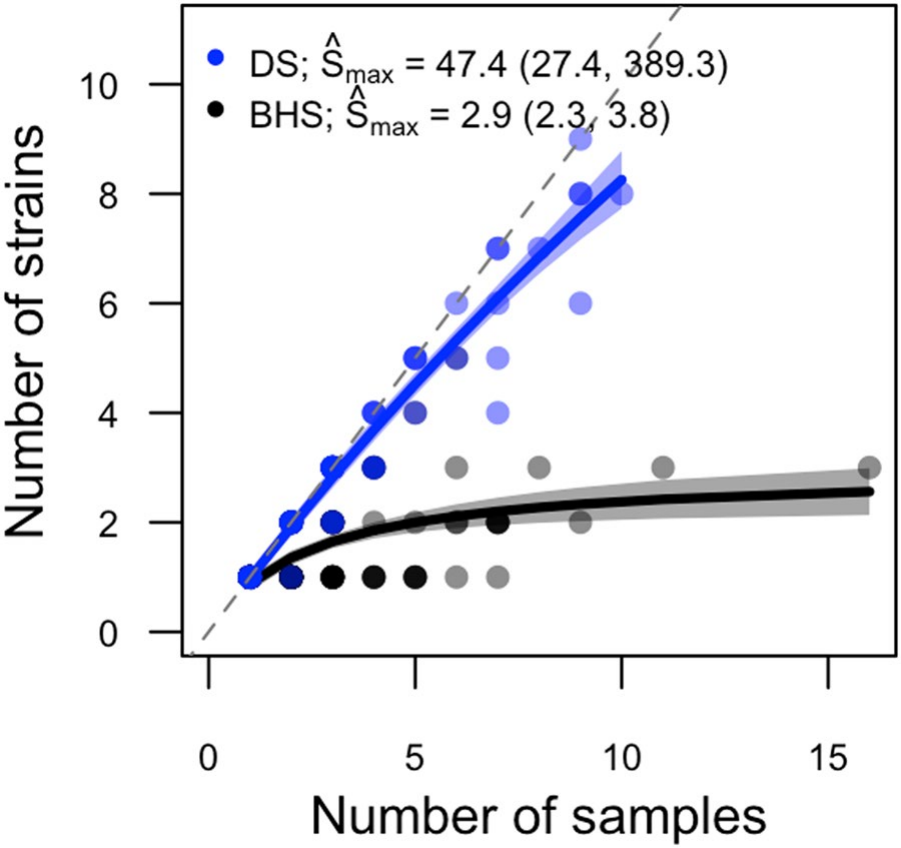
Rarefaction of *Mycoplasma ovipneumoniae* strains found in bighorn (black, BHS) and domestic sheep (blue, DS). Analyses were conducted at the population/operation level.

Non-linear least squares estimates of the strain accumulation curve at the operation- or herd-level were $\hat{B}$ = 47.4 (95% CI [24.8, 437.3]) and ${\hat{S}}_{max}$ = 47.4 ([27.4, 389.3]) for domestic sheep, and $\hat{B}$ = 2.3 ([1.3, 4.0]) and ${\hat{S}}_{max}$ = 2.9 ([2.3, 3.8]) for bighorn sheep. The fitted strain accumulation curve for domestic sheep predicts up to 47 strains may be found within a single herd, and indicates that high levels of sampling would be required to capture the full extent of *M. ovipneumoniae* strain diversity within domestic sheep. In contrast, bighorn sheep within-herd diversity asymptotes at a maximum of approximately 3 strains (Fig. 3).

### Recombination and phylogenetic model selection

A full exploratory scan for recombination in RDP v.4.83 revealed no strong support for recombination within any of the four loci. Of 3 putative recombination events detected within the IGS locus, none could be validated by at least 3 (out of 7) methods (Table S5). Similarly, only a single method detected recombination within *rpoB* and *gyrB*, each; thus, there were no verified recombination events in these loci. No recombination signals were detected within the 16S locus. However, we did detect the possibility of a significant degree of inter-locus recombination, with 38 unique signals of recombination confirmed by at least 3 methods (Table S5). Given this result, we ran the phylogenetic analyses using an alignment with the recombinant sequences removed. Bayesian MLE model selection identified the TN93 model^36^ with gamma-distributed rate variation (TN93 + Γ) as the best fit nucleotide substitution model for the IGS and *gyrB* loci, and the General Time Reversible model^37^ with gamma-distributed rate variation (GTR + Γ) as best fit for the 16S and *rpoB* loci.

### Phylogenetic relationships among *M. ovipneumoniae* strains from wild and domestic hosts

**Figure 4:**
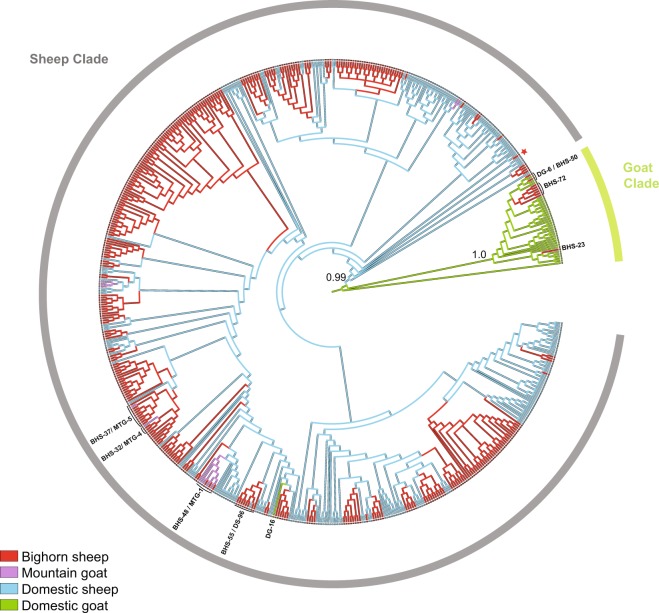
*Mycoplasma ovipneumoniae* consensus tree with predicted ancestral host state traces shown across branches. Phylogenetic analyses were run using all available data, without de-duplication. Posterior probabilities for the two major clades (sheep, goat) are shown. Taxon labels are color-coded by host species (red = bighorn sheep, BHS; purple = mountain goat, MTG; blue = domestic sheep, DS; light green = domestic goat, DG). The red star represents a *M. ovipneumoniae* isolate derived from a bighorn sheep outside of its native range, in a Wisconsin zoo. Bolded text indicates identification numbers for strains found in multiple host species or referred to in the text. See Dataset 1 for complete list of strains and clade assignments.

**Figure 5:**
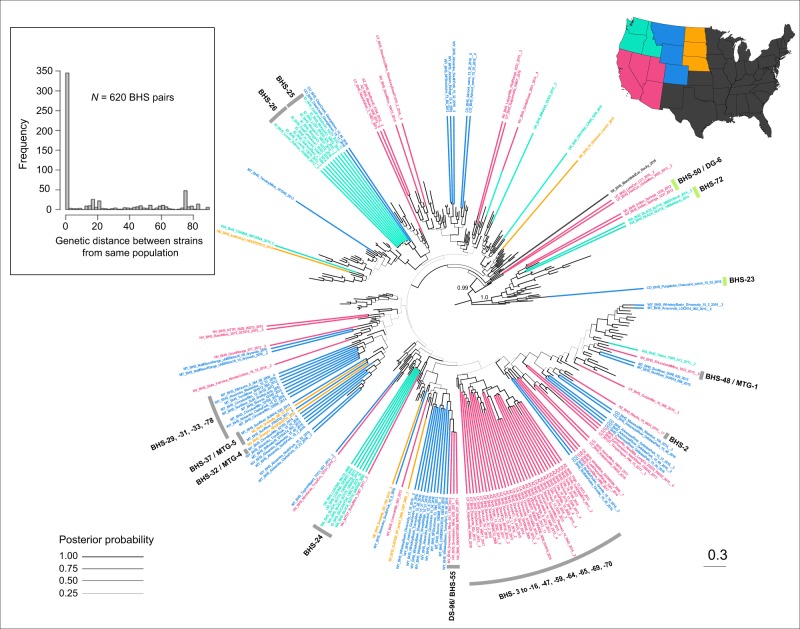
*Mycoplasma ovipneumoniae* consensus tree showing the geographic distribution of strains in bighorn sheep. Taxon are color-coded by region (mint green = northwest, pink = southwest, blue = mountain west, orange = central plains), or are shown in black for strains found outside of the bighorn sheep native range. Domestic sheep, domestic goats, and mountain goats appear as unlabeled branches in the phylogeny. Posterior probabilities are represented by branch width, with thickness relative to probability. Black bolded text indicates identification numbers for strains referred to in the text. Panel inset shows the frequency distribution of genetic distance (i.e., number of mutational differences) for all pairwise comparisons of bighorn sheep strains found in the same location, where geographic distance between pairs = 0.

The *M. ovipneumoniae* consensus phylogeny showed strong support (Posterior Probability (PP) = 1.0) for a domestic goat clade that was highly divergent from the majority of wildlife and domestic sheep strains (Figs 4, S1; light green). The goat clade also included all goat-origin isolates from China. Only one strain detected in a goat (DG-16) collected in Challis, Idaho in 2016, fell outside of this cluster. Nine isolates from bighorn sheep representing three strains (BHS-23, −50, −72) from independent sites in CO, NV and WA also fell within the goat clade, suggesting a minimum of three potential spillover events from domestic goats to bighorn sheep. All domestic sheep, including the Y98 reference strain, the majority of bighorn sheep, and all 12 mountain goat isolates were found in a second major clade (Figs 4, S1, gray), with strains from different species interspersed throughout the clade, indicating the occurrence of multiple transmission events among the three host species. The PPs were relatively low (PP <0.80) for many of the ancestral nodes of the tree, but many sub-clusters within the sheep clade were well supported (PP >0.95), particularly those representing *M. ovipneumoniae* emergence and evolution within bighorn sheep meta-populations (Figs 5, S1).

### Reconstruction of ancestral host states

Ancestral state reconstruction predicted domestic sheep as the most probable host state for ancestral nodes in the “sheep clade,” whereas domestic goats were predicted as the host state for ancestral nodes within the “goat clade” (Fig. 4). Host state changes across the phylogeny was greatest from domestic sheep to bighorn sheep (mean = 35.1, range = 27–42 host state changes; Table 1). Fewer host state changes were observed in the reverse direction, from bighorn to domestic sheep (mean = 10.9, range = 4–19). Host transitions were estimated to be very low (mean = 1–4) from domestic sheep to goats, domestic goats to sheep, and between wild Caprinae species; whereas, no transitions (mean = 0) were estimated from bighorn sheep to domestic goats, mountain goats to domestic sheep, or between domestic goats and mountain goats (Table 1).

**Table 1:** Summary of host state changes along the *M. ovipneumoniae* phylogeny based on ancestral state reconstruction using a parsimony model.

From	To	Min	Max	Mean
BHS	MTG	2	2	2.0
BHS	DS	4	19	10.9
BHS	DG	0	3	0.0
MTG	BHS	3	3	3.0
MTG	DS	0	0	0.0
MTG	DG	0	0	0.0
DS	BHS	27	42	35.1
DS	MTG	3	3	3.0
DS	DG	1	1	1.0
DG	BHS	4	4	4.0
DG	MTG	0	0	0.0
DG	DS	1	1	1.0

The mean, minimum, and maximum number of host changes, “from” the ancestral “to” the derived host state, is shown for each host combination: bighorn sheep (BHS), mountain goat (MTG), domestic sheep (DS), and domestic goat (DG).

### Spatial structure of *M. ovipneumoniae* strains in wildlife hosts

**Figure 6:**
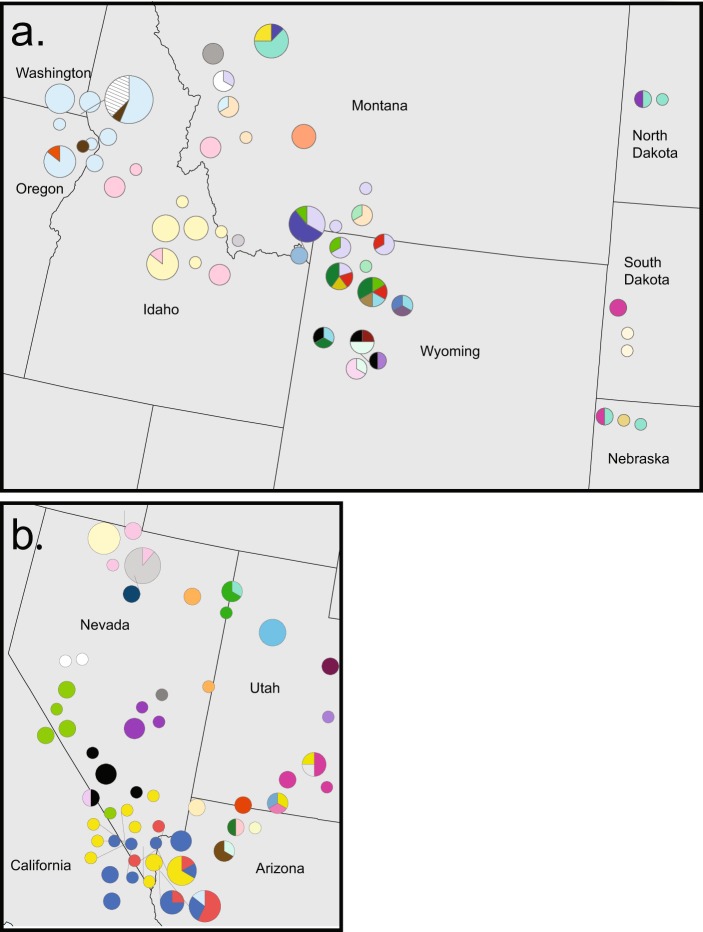
*Mycoplasma ovipneumoniae* strains in bighorn sheep populations. Selected regions include a large proportion of the range of the (**a**) Rocky Mountain bighorn (*O. c. canadensis*) and (**b**) Nelson Desert bighorn (*O. c. nelsoni*) subspecies. Nearly all strains were different between the two regions shown in the panels above, except for one strain (BHS-37/MTG-5; cyan) found in Montana, North Dakota, Nebraska (panel a), and Utah (panel b). With the exception of this strain, colors used in the two maps are independent (i.e., the same or similar color across maps does not indicate identical strains). States not shown similarly exhibited high strain allelic diversity with some strain sharing across neighboring populations.

The *M. ovipneumoniae* phylogeny revealed high phylogenetic diversity within geographic regions. Related strains in bighorn sheep tend to cluster by geography, with identical strains often (~94% of strains) observed only within the same population or neighboring populations (Figs 5 and 6). The most prevalent strain (BHS-24) was detected in 30 bighorn sheep, distributed among 8 spatially proximal populations in Hells Canyon, which spanned Oregon, Idaho, and Washington (Figs 5 and 6a; light blue). Similarly, 17 bighorn sheep shared a strain (BHS-2) across 10 populations in the desert bighorn sheep range (Figs 5 and 6b; blue), and 31 sheep shared two closely related strains (BHS-25, BHS-26) across more than 10 populations in Idaho, Montana, and Oregon (Figs 5 and 6a; light yellow, pink).

Closely related localized strains also illustrate the potential for strain emergence and evolution in bighorn sheep following spillover, examples of which we observed in desert bighorn sheep (BHS-3 to −16, −47, −59, −64, −64, −69, −70) and the Rocky Mountain bighorn sheep across the Greater Yellowstone Ecosystem (BHS- 29, −31, −33, −78; Fig. 5). However, we also observed genetically divergent strains within some populations (Fig. 6), likely representing multiple pathogen introductions. At broader spatial scales, strains in bighorn sheep were nearly always completely different than those found in bighorn sheep from another region (e.g., Fig. 6).

A large proportion (5 out of 12) of mountain goats were from the East Humboldt and Ruby Mountains in Nevada and were infected with a single *M. ovipneumoniae* strain (MTG-1) that was identical to a strain observed in three bighorn sheep (BHS-48) from the East Humboldt Mountains and the Snake Range in Nevada (Fig. 6b, orange). Similarly, a single mountain goat strain (MTG-4) from Castle Creek near Tom Miner, Montana, was shared with a strain identified in three bighorn sheep (BHS-32) collected in nearby Cinnabar Mountain and across the border in northwest Wyoming (Fig. 6a, bright green). Finally, a strain found in a mountain goat (MTG-5) sampled in Battle Creek, South Dakota, was identical to a strain found in 10 bighorn sheep (BHS-37) sampled across populations in North Dakota, Nebraska, Utah, and Montana (Fig. 6a, cyan).

## Discussion

The high degree of genetic diversity of *M. ovipneumoniae* in domestic sheep suggests that the pathogen is likely endemic and that domestic sheep are an important reservoir host and source of infection. In contrast, *M. ovipneumoniae* genetic diversity in wild sheep and mountain goats is low, consistent with a limited number of spillover infections. Ancestral state reconstruction confirmed domestic sheep as a primary source of infection to bighorn sheep, with the highest number of host state transitions (mean = 35) from domestic to bighorn sheep over the pathogen phylogeny. We observed geographical clustering of select strains in bighorn sheep, as well as clusters of related strains that are likely a consequence of intraspecific transmission, persistence, and evolution, following spillover. In addition, we detected multiple distinct strain types within some bighorn populations, which may represent unique spillover events. In contrast, there was little spatial clustering of the diverse strains detected in domestic sheep. Together, these data are most consistent with the occurrence of multiple invasions of *M. ovipneumoniae* from domestic hosts, particularly domestic sheep, into wild Caprinae, and in some cases, pathogen spread and evolution within bighorn sheep following spillover.

*M. ovipneumoniae* detected in goats were genetically divergent from sheep-derived strains, indicating that domestic goats operate as a distinct epidemiological host group, which corroborates previous studies^38^ and supports host-pathogen adaptation in the domestic hosts. Domestic goats were also a source of infection to bighorn sheep, but to a lesser extent than domestic sheep. In contrast, strains detected in mountain goats were all of domestic sheep origin.

### *Mycoplasma ovipneumoniae* strain diversity and the detection of spillover events

High levels of *Mycoplasma ovipneumoniae* genetic diversity, as documented in this study, have also been reported in domestic sheep operations in the United Kingdom^39^, New Zealand^40^, and Iceland^41^. Furthermore, our rarefaction analysis revealed a large difference in the maximum number of strains predicted to be found within domestic versus bighorn sheep herds, and highlights the fact that we are not even close to capturing all the *M. ovipneumoniae* strain types present within domestic sheep operations in the western U.S. Given these observations, it is important to note that our ability to detect spillover events may be limited due to undersampling of domestic host strains. For example, a herd of only 10 domestic sheep is likely to have at least 8 different strains, indicating that even sampling half the herd may miss a spillover strain. We further hypothesize that spillover may be occurring at a higher rate than expected as strains may go undetected if they are not associated with disease events, if they fail to persist due to local extinctions prior to diagnosis, or if there are limited strains that are able to transmit effectively in bighorn sheep. Regular surveillance of bighorn sheep populations combined with more thorough sampling from neighboring domestic operations would be required to better understand the frequency and duration of spillover. Currently, there is no surveillance system for *M. ovipneumoniae* in domestic sheep operations, and disease management strategies for wild Caprinae differ by state. Historically, sampling was opportunistic in bighorn sheep, and primarily occurred during or after an outbreak. Recently, however, many states have initiated statewide *M. ovipneumoniae* sampling of all bighorn herds, regardless of apparent disease states, which may lead to new insights about spillover frequency in the near future.

### Mechanisms promoting spillover and transmission of *Mycoplasma ovipneumoniae*

Domestic and bighorn sheep are closely related, sharing a common ancestor approximately 3 million years ago^42,43^, with a high degree of genome synteny^44^. Similarity in host genetic ancestry may facilitate pathogen spillover^45^ and this is backed by our phylogenetic analysis, which revealed the majority of bighorn *M. ovipneumoniae* strains were most closely related to those from domestic sheep.

Genetic adaptation in both the host and pathogen may also affect the ability to resist infection, thereby influencing the probability of disease emergence within spillover hosts. For example, bighorn sheep experimentally exposed to domestic goat strains of *M. ovipneumoniae* exhibited less severe (non-fatal) pneumonia than has been reported for domestic sheep strains^22,23^. Of the three goat-clade strains detected in bighorn sheep in the present study, only one was associated with observed pneumonia-induced mortality^46^. No conclusions about disease presence or severity were possible in other cases. Wide variation has also been observed in the prevalence and severity of disease associated with spillover of sheep-clade strains into bighorn sheep, some of which may be associated with *M. ovipneumoniae* phylogeny^47^. Furthermore, sequential introductions of different *M. ovipneumoniae* strains within a bighorn sheep population resulted in repeated severe disease outbreaks, suggesting a lack of cross-strain immunity^46^. While analysis of virulence associated with phylogeny is beyond the scope of the present study, it represents a potentially key important area for future research.

We observed mountain goat *M. ovipneumoniae* strains that fell within the sheep clade, and bighorn strains within both sheep and goat clades. These results suggest other factors, such as spatial overlap and the probability of contact, may play a large role in facilitating pathogen spillover. Transmission through contact between domestic and wild hosts is plausible; for example, animals were reported to have escaped their enclosures in 78% of *M. ovipneumoniae*-infected domestic sheep and goat flocks found in close proximity to bighorn sheep^48^. Bighorn sheep herds in proximity to domestic sheep grazing allotments were also more likely to experience a pneumonia-related die-off event^28^. Furthermore, the use of domestic sheep and goats for weed control management was associated with increased risk of a pneumonia epizootic in nearby or overlapping bighorn sheep herds^27^.

### Transmission of *M. ovipneumoniae* in wild Caprinae populations

Emergence of pneumonia in previously healthy bighorn sheep populations presents with a characteristic spatiotemporal pattern of disease: an initial outbreak of fatal pneumonia affecting all age classes, followed by persistent or recurring pneumonia outbreaks, particularly in bighorn lambs, for years or decades afterwards^14^. *M. ovipneumoniae* strain types provide a tool to document this dynamic of invasion, persistence, and disease association, as well as onward transmission to neighboring bighorn sheep populations over extended periods of time. For example, we observed one *M. ovipneumoniae* genotype (BHS-24) in the Hells Canyon meta-population over an 11-year window, between 2006 and 2015. BHS-24 shares identical IGS-, LM- and *gyrB-*locus alleles first detected in an outbreak affecting all age classes in northern Hells Canyon in 1995/96, which suggests this strain was introduced as early as 1995 and has persisted (with ongoing association with respiratory diseases) for 20 years. Similarly, multiple instances of identical or closely-related strains were observed to persist between 2009 and 2017 in neighboring populations across the desert bighorn sheep range in Arizona, California and Nevada^10,49^. Introduction of at least one novel strain after 2011 was associated with more severe disease, and apparent displacement of previous strains^49^. In the desert bighorn metapopulation, some strains appear to have accumulated mutations, again possibly representing transmission, persistence, and strain evolution within bighorn sheep, following a single spillover event. We note that our observation of strain persistence in these particular study sites may be due to increased sampling intensity and could represent a larger phenomenon that may frequently go undetected in populations subjected to less intensive or prolonged pathogen surveillance. Pathogen surveillance can also inform investigations of animal movement and gene flow. When identical *M. ovipneumoniae* strains were found in two bighorn sheep populations previously found to be genetically distinct, presumably due to separation by a landscape barrier^50^, a subsequent genetic study found evidence to support newly increased host gene flow across this barrier^51^.

We observed the sharing of identical strains between sympatric bighorn sheep and mountain goats, indicating some degree of cross-species transmission among wildlife hosts. However, the degree and direction of transmission remains unclear, and dynamics of the disease in mountain goats, until recently, has been largely overlooked. In the East Humboldt Range and Ruby Mountains of Nevada, *M. ovipneumoniae* infections and signs of respiratory disease were documented in mountain goats simultaneous to outbreaks in sympatric bighorn sheep, which resulted in decreased kid survival^52,53^. Strain typing in this region identified mountain goats as a source of infection to reintroduced naïve bighorn sheep following close associations between the two species^54^, data that are represented in our study as strain MTG-1/BHS-48. Previously exposed bighorn sheep and mountain goats have also been documented as asymptomatic carriers, and may play a role in disease maintenance within populations^55^. Our limited data from mountain goats, however, are insufficient to assess the extent of cross-species transmission among wild hosts. Further localized sampling from sympatric bighorn sheep and mountain goats would help to elucidate the transmission dynamics between the two wildlife species.

Our data showing strain-sharing among neighboring bighorn populations most likely represents intraspecific transmission. However, it is important to note that repeated spillover from contact with common domestic sources might also produce this pattern. We suspect this alternative to be less likely given the extraordinary diversity of strains found within a single domestic sheep operation.

### Translocations and disease risk

Translocations have played a vital role in the restoration of bighorn sheep populations across the western United States^56^. However, these well-intended efforts may also have contributed to the movement of pathogen strains to new locations, increasing the risk of disease in naïve populations^35^. From the early 1920s to 2015, there were approximately 1,460 translocation events that involved the movement of over 21,000 sheep in the United States and Canada^56^. For example, the Sun River bighorn sheep population in Montana has frequently been used as a stock population for reintroductions of bighorn within Montana and beyond, including populations in Idaho, Nebraska, North Dakota, Utah, and Washington^57,58^.

While we could not formally assess the effects of translocation on *M. ovipneumoniae* transmission, we speculate that the rare pathogen genetic linkages among geographically disparate bighorn populations may be largely due to management-driven movements of bighorn sheep. We identified two *M. ovipneumoniae* strains associated with the Sun River population (BHS-31, BHS-37/MTG-5) that were geographically dispersed and shared with bighorn sheep and mountain goat in Montana, Nebraska, North Dakota, South Dakota and Utah. However, we are unable to directly track and confirm that these observations of strain sharing are the result of translocations between populations due to the large numbers of bighorn sheep translocated within and among these states over the past five decades.

### MLST approach: Strengths and limitations

This study applied a multi-locus sequence typing (MLST) approach targeting four genetic loci. MLST schemes are widely applied to genetic-based epidemiologic and evolutionary investigations of bacterial pathogens, and have proven useful for studies focused on several *Mycoplasma* species, including *M. agalactiae*, *M. bovis*, *M. hyopneumoniae*, *M. hyorhinis*, *M. mycoides*, *M. pneumoniae*, and *M. synoviae*^59–66^. For *M. pneumoniae*, a MLST assay had increased discriminatory power over traditional typing methods for the detection of distinct strain types and identification of epidemic infection cycles^63^. In addition, the approach has been shown to be valuable for examining the evolution of *M. bovis* strains over time^66^.

One assumption of the MLST approach is that the targeted genetic loci accurately represent the genomic variation in *M. ovipneumoniae*. We acknowledge that our strain typing and phylogenetic reconstructions could be largely influenced by the variation, and therefore discriminatory ability, of the loci included in the MLST assay. Hence, the sequencing of additional loci may enable the discovery of more strains within our dataset or better topological resolution of the phylogenetic relationships among strains. One study on the poultry pathogen, *M. synoviae*, found that while a MLST scheme (based on 7 housekeeping genes) identified the same number of strain types as a conventional single locus assay, it did provide better phylogenetic resolution to aid in the identification of epidemiologically-linked infections^65^.

*Mycoplasma ovipneumoniae* diagnostics based on culture-independent PCR assays were also used in our study, which limited our ability to identify cases of individuals being infected by multiple strains simultaneously, as has been reported to occur in domestic sheep^67^. Also, using this approach, we assumed the amplified sequences for each of the four loci originated from the same strain. Theoretically, if individuals harbor mixed infections consisting of different strain types, the PCR-based MLST approach could result in the false concatenation of loci from different strains, which may misleadingly increase the apparent within-population diversity. However, we only infrequently observed ambiguous sequences consistent with co-amplification of multiple strains, and while we did detect some possible evidence for false concatenation of loci from different strains based on inter-locus recombination events, the number of these possible potentially spurious recombination events were small and our analyses showed that removal of the data associated with these did not affect the conclusions of our study.

## Conclusions

This large-scale investigation into the genetic structure of the primary causative agent of bronchopneumonia across sympatric wild and domestic Caprinae host species provides key insights into pathogen transmission pathways. The genetic data identify domestic sheep as an infection reservoir with multiple and ongoing spillovers to bighorn sheep. Domestic goats are also a source of infection to bighorn sheep, but dynamics of spillover appear to differ from domestic sheep. Strain-sharing across bighorn sheep populations and between wild hosts suggests that, following spillover, pathogen persistence and host movements also contribute to pathogen spread. The ability for *M. ovipneumoniae* to persist and maintain virulence in the absence of spillover is unclear. In addition, we stress that the severity of a pneumonia outbreak and the extent of pathogen spread may be influenced by a combination of strain type, reservoir host species, spillover host immunity, and population exposure history. This knowledge of pathogen movement, invasion frequency, and sources, integrated with data on host-resistant genotypes^68^, will be an informative next step towards predicting the ability of the spillover host species to persist and recover from pathogen invasions.

## Materials and Methods

### Sampling and detection of *Mycoplasma ovipneumoniae*

We obtained 594 samples from *M. ovipneumoniae-*infected bighorn sheep (*n* = 349), mountain goats (*n* = 12), domestic sheep (*n* = 207), and domestic goats (*n* = 26) that were submitted between 1984 and 2017 to the Washington Animal Disease and Diagnostic Laboratory (WADDL) for diagnostic testing or for research purposes (Table S1; Dataset 1). Sample types submitted varied widely: from live animals, they were predominantly submitted as nasal swabs, but from sick animals or necropsy cases included pneumonic lung tissue and/or swabs of the bronchi, trachea, sinus linings or middle ears. Domestic sheep were sampled by the USDA-APHIS-VS National Animal Health Monitoring System (NAHMS) during a survey conducted in 2011, as detailed in Manlove *et al*.^20^.

All sample collection for this study was carried out in accordance with the recommendations in the Guide for the Care and Use of Laboratory Animals of the National Institutes of Health and in conformance with United States Department of Agriculture animal research guidelines, under protocols #03793 and #04482, approved by the Washington State University Institutional Animal Care and Use Committee.

Samples were originated from 19 states (AZ, CA, CO, ID, KS, MI, MN, MT, ND, NE, NM, NV, OR, SD, TX, UT, WA, WI, WY), with isolates spanning the extent of the current bighorn sheep distribution in the western United States (Fig. 1), defined by the Wild Sheep Working Group as the geographic area currently occupied by bighorn sheep. Population origin or herd locations were known for all samples from wildlife and domestic goats, but for only some domestic sheep samples. The majority of the domestic sheep sequence data used were obtained from GenBank (Accession Nos.: MH042304-MH042516, MH045511-MH045514, MH087248-MH087420, MH107389-MH107763)^20^ and only regional localizations (East, Central, or West) within the United States were available. Additional reference strains were included from Australia (*n* = 1, Y98, domestic sheep, 1976, NCBI BioProject PRJNA253514) and China (*n* = 8, domestic goat, 2010, NCBI BioProjects PRJNA253501-4, PRJNA253506, PRJNA253509, PRJNA253511, PRJNA63641).

### DNA extraction and strain typing

The majority of DNA extracts were obtained through the laboratory that performed the original diagnostic testing, predominantly from WADDL, but also included approximately 50 bighorn sheep extracts prepared by the Wyoming Game and Fish Department’s Wildlife Health Laboratory (Laramie, WY). The presence of *M. ovipneumoniae* was detected by previously described polymerase chain reaction (PCR) methods^20,46,69,70^ applied to DNA extracted either from broth culture media or from DNA extracted directly from nasal or lung swab samples. Additional diagnostic samples obtained directly for this study were handled as follows: whole genomic DNA was extracted from *M. ovipneumoniae* broth cultures or swabs using DNeasy blood and tissue kits (Qiagen Inc., Germantown, MD), following manufacturer’s instructions. PCR-positive *M. ovipneumoniae* extracts were genotyped using a multi-locus sequence typing (MLST) approach that targets four genetic loci. The targeted loci are partial DNA sequences from the 16S-23S intergenic spacer region (IGS), the small ribosomal subunit (16S), and housekeeping genes encoding RNA polymerase B (*rpoB*) and gyrase B (*gyrB*). Protocols and primers for PCR amplification of these loci were described previously^46,69,70^. DNA sequencing of amplified PCR products was conducted by commercial service laboratories, including Amplicon Express (Pullman, WA) and Eurofins Genomics (Louisville, KY) for bidirectional Sanger sequencing, and using the same primers used in PCR reactions. All nucleotide sequence ambiguities were coded following the standard codes defined by the International Union of Pure and Applied Chemistry^71^. We aligned sequences for each independent locus in MUSCLE^72^, using default parameter settings as implemented in Geneious R10.1.3 (http://geneious.com, Biomatters, Ltd.).

### Genetic diversity of *Mycoplasma ovipneumoniae* strains by host species

We estimated the genetic diversity of *M. ovipneumoniae* in bighorn sheep, domestic goats, and mountain goats by state and nationally using DNAsp v5^73^, and from domestic sheep regionally and nationally, since state of origin data were not available. Estimated diversity indices include allelic diversity (*A;* the number of different alleles detected), haplotype diversity (*H*_d_; the probability that two randomly sampled alleels are different^74^), and nucleotide diversity (*π*; the mean number of nucleotide differences in pairwise comparisons of DNA sequences^74^). Sequences with ambiguities were excluded from the analysis (9 from bighorn, 29 from domestic sheep), and *H*_d_ and *π* were only estimated for states or regions with at least 5 sequences from a given species, which implies detection of strains present at a minimum frequency of 20%.

### Definition of a strain and rarefaction analyses

Strains that differed by no greater than 4 base pairs (bp) were considered to be the same strain. This was equivalent to ≥99.8% identical sites across the 1,778 bp concatenated sequence alignment. This strain definition was determined after observing a bimodal frequency distribution of pairwise genetic distances in bighorn sheep, and estimating the cutoff between modes to be 5.68 bp in bighorn and 4.90 bp in domestic sheep (Fig. S2). Variants within a strain were uncommon, and the majority of sequences assigned to a strain were 100% identical across nucleotide sites. Strain data were used to generate a rarefaction curve using the individual-based methods outlined by Colwell & Coddington (1994)^75^ and Gotelli & Colwell (2001)^76^. In this model, the cumulative number of strain types, *S(n)*, is treated as a saturating function of the total number of individuals sampled, *n*. Saturation occurs at a rate determined by the total number of strains, *S*_*max*_, as well as a rate parameter, *B*, so that *S(n)* = (*S*_*max*_ *n*)/(*B* + *n*). We used the data to estimate parameters for the Michaelis-Menten-like hyperbolic fit^75,77^. Parameters were estimated using non-linear least squares (R function nls() in package stats), and confidence intervals were based on profile likelihoods.

### Recombination and phylogenetic analyses

Recombination within each locus was assessed using the RDP^78^, GENECONV^79^, BOOTSCAN^80^, MAXCHI^81^, CHIMAERA^82^, SISSCAN^83^, and 3SEQ^84^ programs in RDP4 v4.83^85^. Evidence for a specific recombination event was based on significant support by at least three out of the seven methods^78^.

Individual locus alignments were trimmed and concatenated, resulting in a total alignment length of 1,778 bp, which included variant indels within the IGS locus. For each locus, the best fit nucleotide substitution models were selected by applying a marginal likelihood estimation (MLE) approach^86^ using generalized stepping stone sampling^87^. Model selection was performed in BEAST v1.8.4^88^; for each locus, we combined the results from two independent runs (300 million generations, sampling the posterior distribution every 10,000 generations) in LogCombiner v1.8.4 and assessed convergence in Tracer v1.6^89^.

Evolutionary relationships among *M. ovipneumoniae* isolates were estimated through Bayesian inference using a Metropolis-coupled Markov chain Monte Carlo analysis in MrBayes v3.2^90^, with branch lengths in substitutions per site. Two independent analyses were run in parallel for 50 million generations, each using 4 chains (3 heated, 1 cold) run in parallel to ensure thorough exploration of the tree parameter space. The cold chain is the primary sampled chain, which accepts incremental steps that increase the likelihood of the tree state; whereas, heated chains explore parameter space more freely and can swap with the cold chain upon sampling a state of higher likelihood. Posterior distributions were sampled every 100 generations and model convergence evaluated by ensuring the standard deviations of the split frequencies approached 0 (<0.05) and the potential scale reduction factor was 1 for all parameters. The consensus tree was estimated using combined posterior trees from the two runs, after discarding the first 25% of trees as “burn-in”. The phylogenetic analyses described above were run with all data (*n* = 603), and then limited to unique strains (*n* = 363), after removing duplicate strains, to reduce the over-representation of bighorn sheep outbreaks and of repeated sampling within intensively studied post-outbreak bighorn sheep populations. We observed structuring of location and host data within the resulting pathogen phylogeny to gain insight into pathways of pathogen movement. We further reconstructed the ancestral states of the host to evaluate pathogen transitions (i.e. spillover) over the evolutionary history of *M. ovipneumoniae*. Ancestral state reconstruction analysis was performed assuming a parsimony model in Mesquite v. 3.6^91^, and based on the consensus phylogeny of all *M. ovipneumoniae* isolates generated in MrBayes^90^. Host state changes were summarized, over 100 mappings, in terms of minimum, maximum, and mean number of changes in host state over the phylogeny.

## Supplementary information

Supplementary Information

Dataset 1

## Data Availability

Sequence data for all isolates used in this study can be obtained through the National Center for Biotechnology Information (NCBI) GenBank repository (https://www.ncbi.nlm.nih.gov/genbank/). GenBank accession numbers and associated metadata for each isolate can be found in Dataset 1.
