# Author Correction: Genetic structure of *Mycoplasma ovipneumoniae* informs pathogen spillover dynamics between domestic and wild Caprinae in the western United States

**DOI:** 10.1038/s41598-020-63944-2

**Published:** 2020-04-22

**Authors:** Pauline L. Kamath, Kezia Manlove, E. Frances Cassirer, Paul C. Cross, Thomas E. Besser

**Affiliations:** 1 School of Food and Agriculture, University of Maine, Orono, ME 04469 USA; 2 U.S. Geological Survey, Northern Rocky Mountain Science Center, Bozeman, MT 59715 USA; 3 Department of Veterinary Microbiology and Pathology, Washington State University, Pullman, WA 99164 USA; 4 Department of Wildland Resources and Ecology Center, Utah State University, Logan, UT 84322 USA; 5 Idaho Department of Fish and Game, Lewiston, ID 83501 USA

Correction to: *Scientific Reports* 10.1038/s41598-019-51444-x, published online 25 October 2019

In the Supplementary Information file ‘Dataset 1’, originally published with this Article, an additional column listing animal ID information was provided. Due to an existing confidentiality agreement, this column has been removed in the ‘Dataset 1’ file that now accompanies the Article.

As a result, in the Materials and Methods section under subheading ‘Sampling and detection of *Mycoplasma ovipneumoniae*’,

“We obtained 594 samples from *M. ovipneumoniae-*infected bighorn sheep (*n*  =  349), mountain goats (*n*  =  12), domestic sheep (*n*  =  207), and domestic goats (*n*  =  26) that were submitted between 1984 and 2017 to the Washington Animal Disease and Diagnostic Laboratory (WADDL) for diagnostic testing or for research purposes (Table S1; Dataset 1). Sample types submitted varied widely: from live animals, they were predominantly submitted as nasal swabs, but from sick animals or necropsy cases included pneumonic lung tissue and/or swabs of the bronchi, trachea, sinus linings or middle ears. Domestic sheep were sampled by the USDA-APHIS-VS National Animal Health Monitoring System (NAHMS) during a survey conducted in 2011, as detailed in Manlove *et al*.^20^.”

now reads:

“We obtained sequence data from 594 samples from *M. ovipneumoniae*-infected bighorn sheep (*n* = 349), mountain goats (*n* = 12), domestic sheep (*n* = 207), and domestic goats (*n* = 26). Some samples were submitted between 1984 and 2017 to the Washington Animal Disease and Diagnostic Laboratory (WADDL) for diagnostic testing or to the Besser Laboratory for research purposes (Table S1; Dataset 1). Data originated from M. *ovipneumoniae* detected in multiple sample types, but the predominant sample type was deep nasal swabs from live, apparently healthy animals. Samples from necropsy cases included pneumonic lung tissue and/or swabs of the bronchi, trachea, sinus linings or middle ears. Many domestic sheep were originally part of a previously published study as detailed in Manlove *et al*.^20^. Other domestic sheep and goat samples were obtained by convenience sampling of private operations by the Besser Laboratory.”

