# Supplementary material for: Genetic structure of Mycoplasma ovipneumoniae informs pathogen spillover dynamics between domestic and wild Caprinae in the western United States
Source: Sci Rep. 2019 Oct 25;9:15318. doi: 10.1038/s41598-019-51444-x (PMC6814754; doi:10.1038/s41598-019-51444-x)
Supplement: Supplementary file 1 — Supplementary Information [file 41598_2019_51444_MOESM1_ESM.docx]

**Genetic structure of *Mycoplasma ovipneumoniae* informs pathogen spillover dynamics between domestic and wild Caprinae in the western United States**

Pauline L. Kamath^1,2*^, Kezia Manlove^3,4^, E. Frances Cassirer^5^, Paul C. Cross^2^, Thomas E. Besser^3^

^1^School of Food and Agriculture, University of Maine, Orono, ME 04469

^2^U.S. Geological Survey, Northern Rocky Mountain Science Center, Bozeman, MT 59715

^3^Department of Veterinary Microbiology and Pathology, Washington State University, Pullman, WA 99164

^4^Department of Wildland Resources and Ecology Center, Utah State University, Logan, UT, 84322

^5^Idaho Department of Fish and Game, Lewiston, ID 83501

***Corresponding author:** E-mail: [pauline.kamath@maine.edu](mailto:pauline.kamath@maine.edu), Tel: +1 207-581-2935

**Supplementary Tables**

**Table S1**. Summary of geographic and temporal distribution of *Mycoplasma ovipneumoniae* isolates from bighorn sheep (BHS), mountain goats (MTG), and domestic goats (DG).

| **Location** | **Host Species** | | | **Years** |
| --- | --- | --- | --- | --- |
|  | *BHS* | *MTG* | *DG* |  |
| AZ | 51 |  |  | 2009-2017 |
| CA | 14 |  | 1 | 2009-2016 |
| CO | 26 |  |  | 2007-2016 |
| ID | 33 |  | 4 | 1984-2016 |
| MT | 42 | 1 |  | 2008-2016 |
| ND | 3 |  |  | 2014 |
| NE | 4 |  |  | 2010-2016 |
| NV | 62 | 7 | 1 | 2010-2017 |
| OR | 15 |  |  | 2006-2017 |
| SD | 4 | 1 |  | 2008-2016 |
| UT | 35 | 3 |  | 2010-2017 |
| WA | 26 |  | 20 | 2006-2015 |
| WI | 1* |  |  | 2011-2016 |
| WY | 33 |  |  | 2011-2016 |
| China |  |  | 8 | 2010 |
| **TOTAL** | 349 | 12 | 34 | 1984-2017 |

* BHS from Wisconsin was from a zoo and not free-ranging.

**Table S2.** *M. ovipneumoniae* overall genetic diversity estimates by species in the United States. Genetic diversity indices are reported in terms of allelic diversity (*A*), haplotype (*H*_d_) and nucleotide (*π*) diversity. Sample size (*n*) and standard deviations (SD) of *H*_d_ and *π* estimates are shown. Samples were excluded from those shown in Tables S1 and S2 if ambiguities were present in the sequence.

| **Host Species** | ***n*** | ***A*** | ***H* (SD)** | ***π* (SD)** |
| --- | --- | --- | --- | --- |
| *Bighorn Sheep* | 341 | 118 | 0.981 (0.003) | 0.0216 (0.0003) |
| *Mountain Goat* | 12 | 5 | 0.788 (0.090) | 0.0192 (0.0026) |
| *Domestic Goat* | 26 | 17 | 0.966 (0.018) | 0.0222 (0.0019) |
| *Domestic Sheep* | 179 | 162 | 0.999 (0.001) | 0.0266 (0.0003) |

**Table S3**. Associated metadata and genetic diversity of *M. ovipneumoniae* isolates from domestic sheep, summarized by region. Regions were defined following the USDA-APHIS Center for Epidemiology and Animal Health (CEAH) designated regions (East = MI, MN, WI; Central = CO, ID, KS, MT, NM, SD, TX, UT, WY; West = CA, NV, OR, WA). One additional historical samples from Australia was included. Overall sample size (*N*), time period of sampling (Years), and sample size after removing ambiguities (*n*) for each region are shown. Genetic diversity indices are reported in terms of allelic diversity (*A*), haplotype (*H*_d_) and nucleotide (*π*) diversity. Standard deviations (SD) of *H*_d_ and *π* estimates are indicated in parentheses.

| **Region** | ***N*** | **Years** | ***n*** | ***A*** | ***H* (SD)*** | ***π* (SD)*** |
| --- | --- | --- | --- | --- | --- | --- |
| East | 28 | 2011 | 24 | 22 | 0.993 (0.014) | 0.0265 (0.0010) |
| Central | 107 | 2008-2017 | 94 | 84 | 0.997 (0.002) | 0.0263 (0.0004) |
| West† | 72 | 2009-2017 | 61 | 56 | 0.997 (0.004) | 0.0272 (0.0005) |
| Australia | 1 | 1976 | 1 | 1 | -- | -- |

* *H*_d_ and *π* diversity indices were estimated only when *n* ≥ 7.

† NV is not covered in the USDA CEAH sheep regions, therefore we included domestic sheep samples from the state in the West region

**Table S4**. *Mycoplasma ovipneumoniae* genetic diversity estimates in bighorn sheep, mountain goat, and domestic goat by state. Genetic diversity indices are reported in terms of allelic diversity (*A*), haplotype (*H*_d_) and nucleotide (*π*) diversity. Sample size (*n*) and standard deviations (SD) of *H*_d_ and *π* estimates are shown. Samples were excluded from those shown in Table S1 if ambiguities were present in the sequence, and *H*_d_ and *π* diversity indices were estimated only when *n* ≥ 5.

| **State** |  | *Bighorn sheep* | | | |  | *Domestic goat* | | | |  | *Mountain goat* | | | |
| --- | --- | --- | --- | --- | --- | --- | --- | --- | --- | --- | --- | --- | --- | --- | --- |
|  |  | ***n*** | ***A*** | ***H_d_* (SD)** | ***π* (SD)** |  | ***n*** | ***A*** | ***H_d_* (SD)** | ***π* (SD)** |  | ***n*** | ***A*** | ***H_d_* (SD)** | ***π* (SD)** |
| **AZ** |  | 50 | 20 | 0.938 (0.017) | 0.020 (0.001) |  | 0 | -- | -- | -- |  | 0 | -- | -- | -- |
| **CA** |  | 14 | 5 | 0.842 (0.051) | 0.014 (0.001) |  | 1 | 1 | -- | -- |  | 0 | -- | -- | -- |
| **CO** |  | 24 | 8 | 0.866 (0.035) | 0.019 (0.002) |  | 0 | -- | -- | -- |  | 0 | -- | -- | -- |
| **ID** |  | 33 | 9 | 0.750 (0.063) | 0.007 (0.002) |  | 4 | 4 | -- | -- |  | 0 | -- | -- | -- |
| **KS** |  | 0 | -- | -- | -- |  | 0 | -- | -- | -- |  | 0 | -- | -- | -- |
| **MI** |  | 0 | -- | -- | -- |  | 0 | -- | -- | -- |  | 0 | -- | -- | -- |
| **MN** |  | 0 | -- | -- | -- |  | 0 | -- | -- | -- |  | 0 | -- | -- | -- |
| **MT** |  | 42 | 17 | 0.941 (0.014) | 0.019 (0.001) |  | 0 | -- | -- | -- |  | 1 | 1 | -- | -- |
| **ND** |  | 3 | 2 | -- | -- |  | 0 | -- | -- | -- |  | 0 | -- | -- | -- |
| **NE** |  | 4 | 4 | -- | -- |  | 0 | -- | -- | -- |  | 0 | -- | -- | -- |
| **NM** |  | 0 | -- | -- | -- |  | 0 | -- | -- | -- |  | 0 | -- | -- | -- |
| **NV** |  | 61 | 20 | 0.925 (0.015) | 0.027 (0.001) |  | 1 | 1 | -- | -- |  | 7 | 2 | 0.476 (0.171) | 0.006 (0.002) |
| **OR** |  | 15 | 7 | 0.781 (0.102) | 0.018 (0.004) |  | 0 | -- | -- | -- |  | 0 | -- | -- | -- |
| **SD** |  | 4 | 2 | -- | -- |  | 0 | -- | -- | -- |  | 1 | 1 | -- | -- |
| **TX** |  | 0 | -- | -- | -- |  | 0 | -- | -- | -- |  | 0 | -- | -- | -- |
| **UT** |  | 33 | 18 | 0.951 (0.019) | 0.024 (0.001) |  | 0 | -- | -- | -- |  | 3 | 1 | -- | -- |
| **WA** |  | 26 | 6 | 0.668 (0.082) | 0.018 (0.003) |  | 20 | 12 | 0.953 (0.025) | 0.019 (0.002) |  | 0 | -- | -- | -- |
| **WI** |  | 1 | 1 | -- | -- |  | 0 | -- | -- | -- |  | 0 | -- | -- | -- |
| **WY** |  | 31 | 18 | 0.955 (0.020) | 0.024 (0.001) |  | 0 | -- | -- | -- |  | 0 | -- | -- | -- |

*BHS sample from Wisconsin zoo

# Table S5. Results from full exploratory scan for recombination in RDP4 v4.83. Number of recombination events within each of the four loci (IGS, 16S, *rpoB, gyrB*) detected by seven different analysis programs are reported. Inter-locus recombination was evaluated using the full alignment (ALL), reduced to unique sequences. Total number of specific recombination events within, verified by at least three programs, is shown.

| **Locus** | **Number of recombination events detected by program** | | | | | | | **Total verified events** |
| --- | --- | --- | --- | --- | --- | --- | --- | --- |
|  | *RDP* | *GENECONV* | *BootScan* | *MaxChi* | *Chimaera* | *Sisccan* | *3Seq* |  |
| *rpoB* | 0 | 0 | 0 | 0 | 0 | 24 | 0 | 0 |
| IGS | 0 | 0 | 0 | 1 | 0 | 1 | 1 | 0 |
| 16S | 0 | 0 | 0 | 0 | 0 | 0 | 0 | 0 |
| *gyrB* | 0 | 0 | 0 | 0 | 0 | 7 | 0 | 0 |
| **ALL** | 4 | 4 | 4 | 51 | 16 | 42 | 54 | **38** |

**Supplementary Figures**


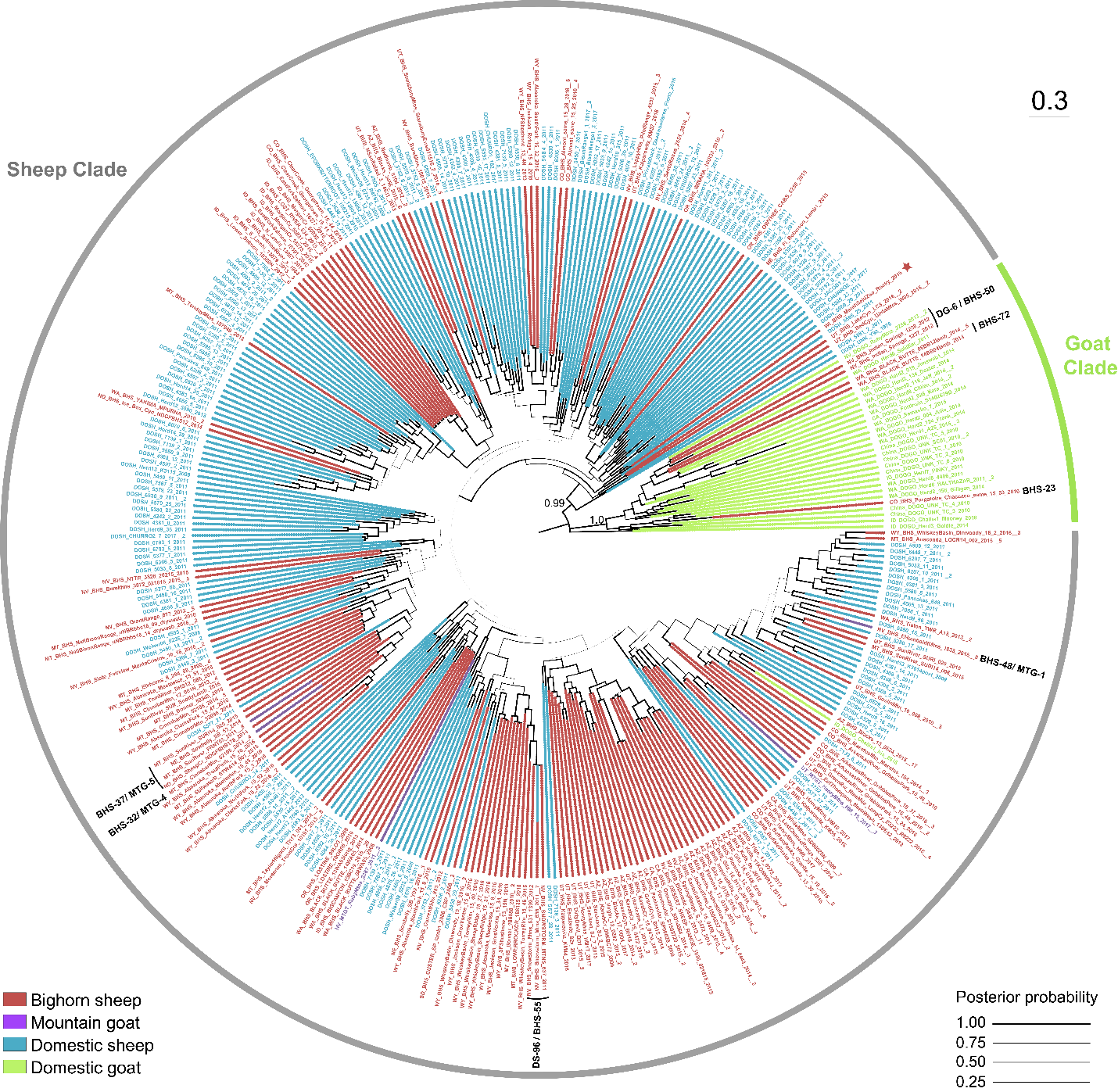


**Figure S1.** *Mycoplasma ovipneumoniae* consensus tree. Identical sequences were removed from the alignment. Taxon labels color-coded by host species (red = bighorn sheep, BHS; purple = mountain goat, MTG; blue = domestic sheep, DS; light green = domestic goat, DG). Posterior probabilities are represented by branch width, with thickness relative to probability. The red star represents a *M. ovipneumoniae* isolate derived from a bighorn sheep outside of its native range, in a Wisconsin zoo. Bolded text indicates identification numbers for strains found in multiple host species as well as those BHS strains that fell within the domestic goat clade (see **Dataset 1**).


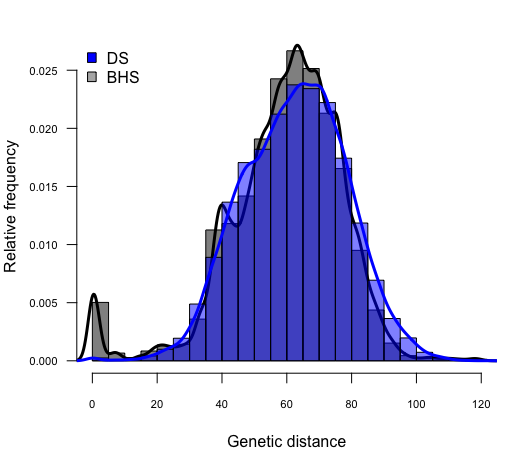
**Figure S2.** Frequency distribution of pairwise genetic distances (in number of single nucleotide polymorphisms) among all pairwise comparisons of strain sequences derived from bighorn sheep (black) and domestic sheep (blue). The solid line indicates local density estimates generated using R’s density function. Assuming a bandwidth of 1.56 (determined using Silverman’s “Rule of Thumb”), we identified a local minimum which we used as a cut-off point separating the two modes. This cut-off was determined to be 5.68 bp in bighorn sheep, and 4.90 bp in domestic sheep (there, using a bandwidth of 1.94). Therefore, we conservatively defined a strain as having no more than 4 bp differences among sequences within the strain.
